# Physical Activity, but Not Glycaemic Load, Is Associated with Lower Real-Time Glycaemic Control in Free-Living Women with Gestational Diabetes Mellitus

**DOI:** 10.3390/nu15081974

**Published:** 2023-04-19

**Authors:** Isabelle R. Jardine, Hannah E. Christie, Kate Oetsch, Angelo Sabag, Meredith Kennedy, Barbara J. Meyer, Monique E. Francois

**Affiliations:** 1 School of Medical, Indigenous and Health Sciences, Faculty of Science, Medicine and Health University of Wollongong, Wollongong, NSW 2522, Australia; 2 Illawarra Health and Medical Research Institute, Wollongong, NSW 2522, Australia; 3 NICM Health Research Institute, Western Sydney University, Westmead, NSW 2145, Australia; 4 Illawarra Shoalhaven Local Health District, Diabetes Service, Wollongong, NSW 2500, Australia; 5 Molecular Horizons, University of Wollongong, Wollongong, NSW 2522, Australia

**Keywords:** pregnancy, exercise, glucose, nutrition, sedentary behaviour

## Abstract

Maintaining blood glucose within the target range is the primary treatment goal for women with gestational diabetes mellitus (GDM). Foods with low glycaemic loads are recommended in clinical practice; however, the relative importance of other key lifestyle variables is unexplored. This pilot study explored the associations of glycaemic load, carbohydrates and physical activity parameters on blood glucose concentrations in free-living women with GDM. Twenty-nine women (28–30 weeks gestation, 34 ± 4 years) with GDM were enrolled. Continuous glucose monitoring, physical activity (ActivPAL inclinometer) and dietary intake and dietary quality were measured concurrently for 3 days. Pearson correlation analyses determined the association between glucose levels and lifestyle variables. Despite all receiving the same nutrition education, only 55% of women were following a low glycaemic load diet with a large range of carbohydrate intakes (97–267 g/day). However, the glycaemic load did not correlate with 3-hr postprandial glucose (*r*^2^ = 0.021, *p* = 0.56) or 24-h glucose iAUC (*r*^2^ = 0.021, *p* = 0.58). A significant relationship between total stepping time and lower 24-h glucose iAUC (*r*^2^ = 0.308, *p* = 0.02) and nocturnal glucose (*r*^2^ = 0.224, *p* = 0.05) was found. In free-living women with diet-controlled GDM, more physical activity, i.e., steps accumulated across the day, may be a simple and effective strategy for improving maternal blood glucose concentrations.

## 1. Introduction

Gestational diabetes mellitus (GDM) is classified as glucose intolerance that is first detected during pregnancy [1] and is commonly diagnosed between 24–28 weeks gestation following an oral glucose tolerance test (OGTT). GDM is the most common complication in pregnancy, with the prevalence increasing in recent years, now afflicting ~20% of pregnancies worldwide [2]. Poor management of GDM leads to a high risk of maternal and neonatal complications, including excessive fetal growth, which may result in adverse perinatal outcomes such as fetal macrosomia and neonatal hypoglycaemia [2,3,4]. GDM also increases the risk of the baby developing childhood obesity and the mother developing type 2 diabetes mellitus later in life [4]. Accordingly, the management of maternal blood glucose concentrations is the primary goal in GDM management.

Medical nutrition therapy (MNT) is the first-line therapy for GDM management [3]. As carbohydrates are the primary macronutrient that increases blood glucose concentrations, MNT focuses on the quality of carbohydrates and their distribution across the day. A diet including mainly foods with a low glycaemic index (GI) or low glycaemic load (GL) is a common recommendation; however, the evidence for this recommendation to improve maternal glucose control and infant outcomes is mixed. For example, with respect to GI, randomised controlled trials (RCTs) show no difference between low or moderate GI diets for maternal glucose or maternal and neonatal outcomes [5,6]. The benefits of a low GI diet are only apparent when compared to a high GI diet [6,7,8]. Outside of RCTs, the effects of GI on glucose outcomes in free-living women with GDM are unknown. However, because GI-focused interventions centre solely on the dietary quality of carbohydrates, GL, which considers both the quality and quantity of the carbohydrates consumed in the diet, is considered a better indicator of overall dietary quality and should also be explored [9,10].

Over the past decade, physical inactivity, including sedentary time, has emerged as an important modifiable risk factor due to its deleterious effects on cardiometabolic morbidity and mortality [11]. Despite little research on the effects of sedentary time on maternal health, regular physical activity prior to and during pregnancy has been shown to lower the risk of developing GDM [12,13]. Although physical activity is critical for improving cardiometabolic health, it is unclear whether a specific volume or intensity is required to see such benefits in a GDM pregnancy. Of interest, a recent study found those achieving more than 6000 steps/day had lower maternal glucose concentrations compared to women with GDM below the 6000 steps/day threshold [14]. Overall, there was a strong relationship between more steps per day having better glucose outcomes [14]. While increasing total physical activity leads to improved cardiometabolic outcomes in pregnancy, research has also found that more breaks in sedentary time (sit-to-stand transitions) across the day are associated with lower fasting and 2-h oral glucose concentrations in women with GDM [15]. Consequently, further research is required to elucidate the relative importance of sedentary behaviour, and physical activity volume and intensity for improving maternal blood glucose concentrations and cardiometabolic outcomes in women with GDM.

Increased physical activity and dietary modulation are considered the cornerstone therapies of GDM management. However, there is limited research available on free-living women undergoing standard care management to determine the relative contribution of each on maternal blood glucose control. Consequently, the primary aim of this study was to determine the relationship between key parameters of dietary intake and physical activity on maternal glucose concentrations in women with GDM. An exploratory aim was to describe the nutrient intake of women with GDM, compare this to Nutrient Reference Values (NRVs) and assess the quality and quantity of carbohydrates consumed by calculating GL. It was hypothesised that physical activity volume (steps/day and stepping time) would be inversely, and GL positively, associated with glucose control in women with diet-controlled GDM.

## 2. Methods and Materials

### 2.1. Participants, Demographics and Ethics Approval

The participants of this study were recruited via Illawarra Shoalhaven Diabetes Service (ISDS) during their first group education visit to the service. To be eligible for this study, participants were required to: be over the age of 18, have a diagnosis of GDM [16], be diet-controlled and be between 28 and 30 weeks gestation. Women were excluded if they were taking insulin or oral hypoglycaemic medications. This cross-sectional study is an exploratory analysis of baseline data collected for a randomized controlled trial (RCT) running from September 2018 and March 2022. Eligible participants provided written informed consent prior to enrolment. The study conformed to the ethical guidelines of the 1975 Declaration of Helsinki, and ethical approval for this study was obtained by the Joint University of Wollongong Illawarra Shoalhaven Local Health District Human Research and Ethics Committees (2018/31, approval date: 7 August 2018).

### 2.2. Dietary Education Session

Upon referral to the ISDS, women attended a group education session where they were educated on the dietary management of GDM, including the quantity, quality and distribution of carbohydrates throughout the day. Specifically, women were advised to consume 3 meals and 3 snacks per day, each containing 30 g of carbohydrates and were educated and encouraged to consume a diet of low GI carbohydrates. Healthy eating advice for pregnancy based on the Australian Guide to Healthy Eating (AGHE) was also provided by an Accredited Practising Dietitian [17]. After the group education session, the women who volunteered for this study were given a 2D food model booklet to assist with portion estimation and asked to complete a 3-day food and beverage record and wear a continuous glucose monitor (CGM) and inclinometer for the same duration (7 days). Any dietary supplements and vitamins taken were noted and included in the micro-nutrient analysis and compared to those who did not take any supplements. To ensure food data recorded by participants were complete, a follow-up interview with each participant was conducted by a student Dietitian (IJ).

### 2.3. Continuous Glucose Monitoring

Women wore a CGM (iPro2 Medtronic MiniMed, Northridge, CA, USA) for 7 days; however, for analysis, 3 full 24-h days were used that corresponded with matched food records for each participant. The CGM is positioned via a microneedle in the subcutaneous tissue on the back of the upper triceps, connected to an adjacent monitor. It measures interstitial fluid glucose levels continuously before using an algorithm to develop and store an average glucose concentration every 5 min [18]. The CGM is linked to a smartphone application that allowed women to record their food data and 4 calibration finger pricks daily (iPro Medtronic App, Medtronic Australasia Pty Ltd., Macquarie Park, Australia). All data was stored until the CGM was removed and downloaded using online Carelink software (Medtronic, Medtronic Australasia Pty Ltd, Macquarie Park, Australia) before raw data was exported for analysis. Mean nocturnal glucose was calculated from 00:00–05:00, and 3-h postprandial glucose was calculated from main mealtimes recorded in food diaries.

### 2.4. Physical Activity and Sedentary Time

An ActivPAL3 inclinometer (PAL Technologies Ltd., Mississauga, ON, Canada) was used to objectively measure physical activity and sedentary time. The ActivPAL inclinometer is a valid and accurate tool to measure incidental and sedentary behaviour in free-living environments [19]. The ActivPAL was placed upright on the anterior, proximal 1/3 of the lower limb and secured with a clear film Tagaderm. The ActivPAL inclinometer calculates physical activity (>20 steps·min^−1^) and sedentary time through changes in posture and movement in 3 planes; sitting (postural changes, no acceleration), standing (posture and acceleration) and stepping time. Daily averages for each variable were calculated using the PAL software (PAL software suite, Version 8, PAL technologies) for the same 3 days that the diet was recorded.

### 2.5. Dietary Intake—Nutrient, Food Groups and Carbohydrate Quality Analysis

Participants recorded all foods and beverages consumed for 3 days (2 weekdays and 1 weekend day) whilst wearing the CGM. The food items were entered into FoodWorks Dietary Analysis Program (v9, 2017. Xyris Software Inc., Highgate Hill, QLD, Australia). FoodWorks nutrient data is based on the complete food composition database AUSNUT 2011-13. Data was exported into Microsoft Excel (Microsoft Office Professional Plus v14.0.7237.5000, 2010. Microsoft Corporation, Washington, DC, USA), where GI and GL values were then calculated for analysis.

### 2.6. GI and GL Calculations

The GI values used were extracted from a special glycaemic index edition of AUSNUT2011–2013 (Glycemic Index Foundation (GIF). AUSNUT 2011–2013 glycemic index edition, GIF: 2015. Glebe, Australia), whose values were based on a previously published method [20].

GL was calculated using the following equation: GL = glycaemic index of individual food item (percentage) multiplied by the amount of carbohydrates available per portion of food (grams (g))/100 (and therefore the unit is in grams) (GL g = GI (%) × CHO g/100). Dietary GL per individual was calculated as the sum of all GL foods reported on each separate day and then divided by 3.

Food items were classed into low, medium or high by the amount of GL the food item contained using the accepted ranges low (≤10 g), medium (11–19 g) and high (≥20 g) [21]. A low GL diet was classified as consuming <100 g per day [20].

### 2.7. Diet Quality

The macro- and micro-nutrient data was assessed against the National Health and Medical Research Council’s (NHMRC) Australian and New Zealand nutrient reference values (NRVs) for pregnancy [22] and were expressed as the mean, standard deviation (SD), range and total percentage of women who met the macro-, micro-nutrient and food group optimal requirements for their applicable age group. Food group intakes are reported as the proportion (%) meeting (±1 serve), exceeding and below the recommendations. Dietary intakes lower than the appropriate NRV were considered inadequate. There are no exact energy NRVs, so energy was not included in the analysis. Water intake was not included in the food records, as fluid intake adequacy was not an aim when analysing nutritional adequacy. Similarly, each food record was compared against the NHMRC’s recommended serves per food group for pregnant women. Basic carbohydrate quality was assessed by calculating a percentage total of the daily carbohydrate intake from low, medium and high GL groups of classification.

### 2.8. Statistical Analysis

As this was an exploratory pilot analysis, no sample size calculations were performed. Descriptive statistical analysis was performed in SPSS (IBM SPSS Statistics v25, 2017. New York, NY, USA), and data were tested for outliers using boxplots. GraphPad Software (GraphPad, Prism v8, 2019. GraphPad Software, San Diego, CA, USA) was used to investigate the relationship between the mean dietary GL consumed per participant and the mean diurnal blood glucose levels (0500–2400); the mean GL consumed at meals only (excluding snacks) with the mean blood glucose levels experienced 3-h postprandial; and the mean dietary GL consumed against the 24-h mean of iAUC; all as an average of 3 days (*n* = 22). Regression analysis confirmed by a Pearson correlation was used to determine the strength and goodness of line fit (*r* square) and significance of results (*p*-value). Hierarchical linear regression analyses were performed for 24 h glucose. Pre-pregnancy BMI was entered into the regression at Block 1. Glycaemic load and carbohydrate g/day were entered into block 2 of the model. The total steps or stepping time were entered into Block 3 of the model. A *p*-value < 0.05 was considered statistically significant.

## 3. Results

### 3.1. Participant Demographics

Twenty-nine pregnant women with GDM were initially recruited; of those, six did not begin or withdrew before data collection, three had incomplete food diaries, and four had missing inclinometer data, leaving 22 complete data sets for GL analyses and 18 for physical activity (ActivPAL) analyses. Women were diet controlled, aged on average 34 ± 4 y with a pre-pregnancy body mass index (BMI) of 26 ± 4 kg/m^2^. Of these, 39% were in their first pregnancy, and of the 61% who were in their second or third pregnancy, 18% of these had been diagnosed with GDM previously. Most women (77.8%) were on pregnancy-supportive nutritional supplements, which included Elevit Pregnancy Multivitamin (Elevit, Bayer Australia Ltd., Gordon, Australia), Blackmore’s Pregnancy and Breast-feeding Gold and folic acid supplements. Other supplements taken in conjunction with these included vitamin D, vitamin C and iron tablets. 16.7% were taking no nutritional supplements at all, and 5.6% were only taking omega-3 supplements and a probiotic. Two women were vegetarians, and the others had no special dietary requirements.

### 3.2. Glycaemic Load: Carbohydrate Quality and Quantity Assessment

An average of 176.3 ± 47.4 g of carbohydrates were consumed per day. The mean daily dietary GL intake was 95.6 ± 48.2 (ranging from 11.5–244.7). The GL for meals and snacks only were 72.3 ± 40.9 and 23.3 ± 19.25, respectively. Fifty-five percent of women were considered to have a low GL diet based on their mean dietary GL intake. Of the total carbohydrate foods, 12.6% of items were classified as high GL, 15.8% as medium GL and 71.6% as low GL. Snacks comprised 33.5% of the total carbohydrate foods consumed across the day. These were sub-categorised into high, medium and low GL foods; 6.4%, 10.6% and 83.1%, respectively.

### 3.3. Relationship between Physical Activity, Sedentary Behaviour and CGM Data

**Figure 1:**
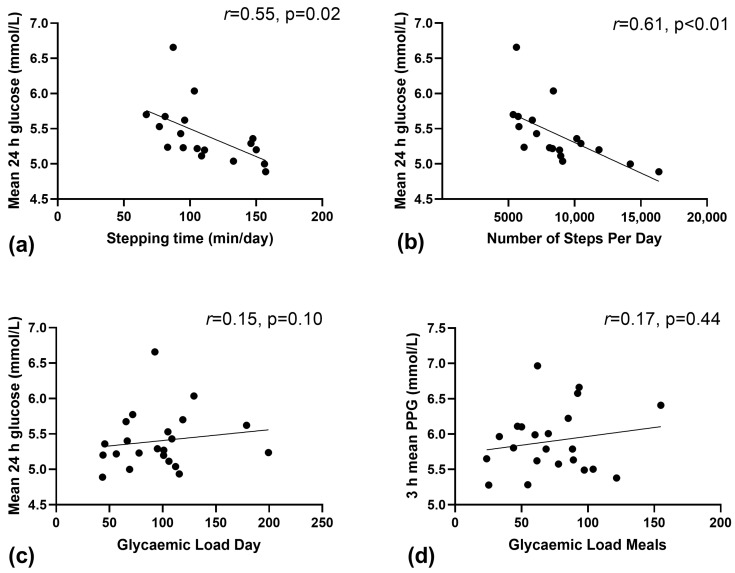
Relationship between continuous glucose derived measures of glucose and physical activity and glycaemic load (GL) in free-living women with GDM. (**a**) Mean 24 h glucose and stepping time, (**b**) Mean 24 h glucose and the number of steps/day, (**c**) Mean 24 h glucose and daily glycaemic load, and (**d**) mean 3-h postprandial blood glucose and glycaemic load of main meals.

On average, women completed 8743 ± 3022 steps/day, spent 111 ± 29 min/day (~1 h 51 min) in stepping time and 1007 ± 173 min/day (~16 h 47 min) in sedentary time. There was a significant inverse relationship between daily stepping time; and mean glucose (Figure 1a, *r*^2^ = 0.308, *p* = 0.02) and nocturnal glucose (*r*^2^ = 0.224, *p* = 0.05). In addition, there was a moderate inverse correlation between the number of steps per day and mean 24 h glucose (Figure 1b, *r*^2^ = 0.383, *p* < 0.01) and nocturnal glucose (*r*^2^ = 0.241, *p* = 0.04). There was no relationship between the total min per day of sedentary time and mean glucose (*r*^2^ = 0.020, *p* = 0.58).

### 3.4. Relationship between GL and Corresponding CGM Data

Figure 1c shows no relationship between daily GL and mean blood glucose (*r*^2^ = 0.023, *p* = 0.50). Similarly, there was no relationship (*r*^2^ = 0.030, *p* = 0.64) between the amount of GL consumed at meals and the mean 3 h postprandial glucose (Figure 1d, *p* = 0.44). No relationship was found between the daily GL and the total 24-h iAUC (*r*^2^ = −0.129, *p* = 0.10), nor was there between grams of carbohydrate consumed per day and postprandial glucose (*r*^2^ = 0.085, *p* = 0.19).

### 3.5. Hierarchical Linear Regression Analyses

Pre-pregnancy BMI accounted for 26% of the variance in 24 h blood glucose concentrations (*p* = 0.03). The inclusion of GL and carbohydrate g/day did not affect blood glucose (26%, *p* = 0.93), but the addition of steps/day increased the prediction to 44%, which was trending towards significance (*p* = 0.07). Stepping time increased the prediction of blood glucose concentrations beyond that of BMI, GL and carbohydrate consumption to 39% but did not reach statistical significance (*p* = 0.12).

### 3.6. Dietary Quality Assessment

#### Macro- and Micro-Nutrient Intake Assessment

The mean energy intake was 8388.4 ± 1842.3 kJ per day, with the range being 6647.9–13,290.6 kJ. Of the mean energy intake, 19.1 ± 3.2% was provided by protein, 37.4 ± 6.7% by carbohydrate, and 39.4 ± 6.0% from fat sources. Additional percentages of mean daily energy were derived from fibre (2.6 ± 0.6%) and other dietary energy sources (1.5 ± 1.1%).

Mean intake per nutrient, relevant ranges and the percentage of women who met the NRVs solely with diet and those who would have met their NRVs with nutritional supplementation are shown in Table 1.

The majority of women did not achieve the estimated average requirement (EAR) for dietary fibre, vitamin B6, iodine and potassium (Table 1). Merely a third of the women were meeting their EAR for dietary fibre. Most women met the EAR for protein, vitamin C, zinc and calcium. Phosphorus adequate intake (AI) was met by all women through dietary intake alone. With the assistance of nutritional supplements, 100% of women were able to reach the appropriate EAR or AI for riboflavin, niacin (equivalents), vitamin C, zinc, selenium and vitamin E (Table 1). All women failed to meet the EAR for iron and so were considered inadequate through diet alone; nevertheless, women taking Elevit as a supplement were able to meet the EAR. Similarly, through diet alone, the folate EAR was exclusively met by approximately half of the women; and with supplementation, 100% of women met the EAR for folate. The mean intake of sodium exceeded the adequate intake by 293% (Table 1).

### 3.7. Australian Guide to Healthy Eating (AGHE) Food Group Assessment

Table 2 depicts the mean number of serves of each food group consumed by women. The food group that most closely aligned with recommended serves was fruit. The food group which had the highest proportion of women exceeding the recommendations was meat and meat alternatives, and the food group with the most women below the recommendations was cereals and grains.

## 4. Discussion

### 4.1. Main Findings

In contrast to our hypothesis, there was no association between GL (overall, meals or snacks) and key CGM-based indices of glucose control (i.e., postprandial, mean 24-h and area under the curve). However, there was a moderate inverse correlation between performing a more incidental daily activity (stepping time, number of steps per day) and mean 24-h and nocturnal (overnight) glucose control. Medical nutrition therapy (MNT) and physical activity are frontline therapies for women with GDM to manage maternal glucose concentrations, particularly postprandial glucose. Controlling postprandial glucose is important as it is strongly related to adverse outcomes, such as macrosomia [23]. GDM management and treatment has a large focus on consuming low GI foods; however, this is largely based on evidence from people with type 2 diabetes, as there is limited research in women with GDM [24]. The findings from this small pilot study support encouraging more steps per day and minutes of stepping as a simple yet underutilised strategy for managing maternal glucose in women with GDM. Though, future randomised clinical trials are warranted.

### 4.2. Increasing the Number of Steps per Day; an Important Physical Activity Recommendation

Physical activity is an important adjunct therapy for women with GDM [25]. However, the importance and effectiveness of physical activity (being active across the day) for managing maternal hyperglycaemia are not always emphasised. Less than half of women with GDM in Australia report meeting the physical activity guidelines for pregnancy [26]. Only 53% of pregnant women in Australia reported receiving some form of exercise advice, and many reported receiving advice that may not be included in the current guidelines for physical activity during pregnancy [27]. There are currently no specific physical activity guidelines for women with GDM. However, in line with the general guidelines for adults, women are encouraged to meet pregnancy physical activity recommendations of at least ~150 min of moderate physical activity and undertake two muscle-strengthening sessions weekly. Outside of these recommendations for physical activity, our findings show that women who were performing more steps/day had lower mean 24 h glucose concentrations, which is in line with previous research by Hayashi et al. [14]. Advising women to increase the number of steps/day may be a simple and cost-effective public health message to improve GDM management. In addition to managing glycemia, previous research has suggested increasing steps/day in early pregnancy may also reduce the risk of developing GDM [28]. In a cross-sectional study undertaken in Norway, women diagnosed with GDM had lower steps in early pregnancy (<20 weeks) compared to women who were not diagnosed with GDM at 28 weeks, where the odds of developing GDM were 19% lower with an increase of ~3000 steps per day [28]. We also found the more minutes women spent stepping was related to lower mean and nocturnal glucose; however, there was no relationship for sitting/sedentary time. Taken together, our findings highlight the potential importance of simple physical activity recommendations, such as increasing the number of steps per day, for lower fasting and postprandial glucose responses in women with GDM.

### 4.3. Carbohydrate and Glycaemic Load

Choosing low glycaemic foods and distributing carbohydrates across the day are key components of MNT for diabetes management. As part of standard care, these women received education on a low GL diet and were recommended to consume ~180 g carbohydrates (as two 15 g carbohydrate exchanges at meals and snacks) distributed evenly across the day. However, only 55% of women were consuming a low GL diet with a large range of carbohydrate intakes (~97–267 g). Despite this large range, there was no clear relationship between the amount of daily carbohydrates consumed or the GL and nocturnal and postprandial glucose concentrations. Although this contrasts with previous research, all prior work has randomised women to either low or high GI diets, and as expected, found that low GI diets improved fasting and postprandial glucose [22], nocturnal glucose, increased time within the target range and decreased glycaemic oscillations [9]. Whereas this study explored the relationship of GL with maternal glucose control in women with GDM under habitual free-living conditions. Given there was no relationship between GL and carbohydrates and glucose control and that steps per day and stepping time are independent predictors of glucose control, our findings highlight the importance of recommending physical activity (specifically the number of steps per day) as frontline for the standard care management and treatment of GDM.

### 4.4. Achieving Nutrient Recommendations

In addition to glycaemic load, diet quality is emphasised in the clinical management of GDM as it has been shown to be important in managing glycemia and reducing the risk of pregnancy complications [29]. In this exploratory analysis, including detailed food records from women, we found most women were inadequate in iron, dietary fibre, vitamin B12, iodine and potassium (Table 1). Only 22.2% of women met recommendations from all food groups; 5.6% using nutritional supplementation met all NRVs and AGHE recommendations, highlighting the importance of supplements in pregnancy, particularly to meet folate and iron EAR [1,2]. Only 56% of women were meeting the folate EAR through diet alone. Considering folate is crucial for fetal development [30], these outcomes are concerning and resonate with the results of preceding studies [2,10]. In the present study, 50% of women who were sufficient in dietary folate had a low GL diet. Main sources of folate in the Australian diet derive from cereals, cereal products, vegetables and legumes, so unsurprisingly, of those who were dietarily deficient in folate, 62.5% were inadequate in grains/cereals and/or vegetables.

Our findings and those of Louie et al. [10] demonstrate that women with GDM are consuming nutritionally inadequate diets. This study provides preliminary data on what women undergoing standard care in real life are consuming, the findings strongly highlighting the need for future research that measures intakes and explores how women can better meet their NRVs whilst on a GDM diet.

### 4.5. Strengths and Limitations

Strengths of this study include the use of real-time CGM data with paired detailed diet records collected in free-living women with diet-controlled GDM. Compared to one-off blood sampling, CGM measures blood glucose every 5–10 min, providing a full glucose profile that permits the ability to more accurately and effectively match postprandial peaks to GL consumed at meals and snacks. Similarly, the use of objective physical activity monitoring using inclinometers provides accurate (without reporting bias) and valuable information on key physical activity parameters (i.e., stepping time, sitting time, steps). These data are also paired alongside real-time CGM in free-living settings. This study provides novel information on the dietary practices of free-living women with GDM post-general nutrition education without an enforced dietary intervention. This is important as a recent survey of women with GDM in Australia found that 88.3% of women reported that they relied on dietary changes to manage their blood glucose concentrations, and the most effective approach reported being lowering the amount of carbohydrates in meals and snacks to best control hyperglycaemia [26].

This study also had its limitations, most notably the small sample size and observational study design. Findings should be confirmed in a large, well-designed RCT. Furthermore, selection bias could be evident as participants were initially recruited for an exercise-based study (which had to be put on hold during the COVID-19 pandemic). Due to their enrolment in an exercise-based trial, these women may be more inclined to undertake a healthier lifestyle than that of the average population with GDM, thus, potentially not reflecting the typical dietary and physical activity habits of women with GDM. Furthermore, this study only captures one point in pregnancy and, therefore, cannot be a true reflection of overall nutritional adequacy during a whole GDM pregnancy. Although an average of 3 days (1 weekend day and 2 weekdays) was used to capture a typical diet, averaging across the day dilutes our ability to compare meal timing, composition and glucose fluctuations.

### 4.6. Summary

This is the first study, to the best of our knowledge, that has explored the relationship of GL (with no enforced dietary intervention) against real-time CGM-derived measures of maternal glycaemia in women with GDM, and, unexpectedly, we found no relationship between GL and glucose outcomes. We did, however, find a correlation for physical activity, where stepping time and number of steps per day were associated with better glucose control outcomes. The results of regression analysis demonstrate that pre-pregnancy BMI accounted for 26% of the variance in 24 h blood glucose concentrations among study participants. When controlling for pre-pregnancy BMI, glycaemic load and total carbohydrates do not associate with 24 h glucose, but stepping time and total steps are independent predictors of 24 h glucose. A strength of this study is the exploration of GL and physical activity in free-living settings, which has high generalisability and translation. Many women are expected, to a large extent, to manage their own blood glucose levels by trial and error following a group education session. Although a combination of diet, physical activity and pharmacological interventions are required to manage GDM—recommendations to simply do more steps per day may be a simple, achievable and effective frontline public health message to improve GDM management. Future intervention studies are now needed.

## 5. Conclusions

In free-living women with GDM, those with higher levels of physical activity through increased steps per day had better continuous blood glucose levels. Most women are not meeting the EAR for various vital nutrients.

## Figures and Tables

**Table 1:** Mean (±SD) and Range of Nutrient Intake of Women in the present study and Percentage of Appropriate Nutrient Reference values Met Based on Pregnant Women Aged 19–50 [22].

Nutrient	Unit/Day	Mean (±SD)	Range: Min–Max	% Meeting NRVs: Diet Only	% Meeting NRVs through Supplements
EAR					
Macronutrients					
Protein	g	92.5 (17.3)	48.9–116.7	94	94
Carbohydrate ^¶^	g	176.3 (47.3)	96.9–266.9	56 ^¶^	56
Dietary Fibre	g	26.8 (6.5)	16.7–40.4	33	33
Vitamins					
Thiamin	mg	1.9 (1.2)	0.7–5.5	67	94
Riboflavin	mg	2.0 (0.50)	0.9–2.9	94	100
Niacin Eq.	mg	21.2 (5.0)	12.6–29.3	89	100
Vit B6	mg	1.5 (0.3)	1.1–2.0	39	83
Vit B12	µg	4.2 (1.2)	2.1–6.4	94	94
Folate	µg	494.5 (134.7)	293.9–723.3	56	89
Vit A	µg	1154.2 (734.8)	299.8–2898.1	83	94
Vit C	mg	94.8 (48.2)	36.5–222.3	94	100
Minerals/Trace Elements					
Calcium	mg	1014.5 (304.6)	419.2–1436.9	83	83
Phosphorus	mg	1576.6 (347.8)	1092.9–2483.5	100	100
Zinc	mg	11.9 (2.8)	8.2–17.2	89	100
Iron	mg	11.0 (2.9)	7.1–17.2	0	39
Iodine	µg	160.1 (38.2)	90.4–219.3	50	78
Selenium	µg	80.4 (31.4)	29.4–172.1	83	100
AI					
Macronutrients					
Polyunsaturated Fatty Acids					
Linoleic Acid (N-6)	g	12.3 (5.1)	5.3–26.4	61	61
A-Linolenic Acid	g	2.0 (0.9)	0.8–3.6	94	94
Omega-3 Long Chain PUFA DHA/EPA/DPA	g	0.2 (0.3)	0.0–1.1	56	72
Vitamins					
Vit E	mg	14.3 (5.7)	5.3–27.3	94	100
Minerals/Trace Elements					
Magnesium	mg	340.6 (90.4)	237.8–588.3	61 ^Ɨ^	83
Potassium	mg	2879.9 (503.3)	2148.2–3726.8	44	44
Sodium	mg	2696.7 (757.2)	1571.1–4314.2	100	100

SD: standard deviation, Min: minimum, Max: maximum, % percentage of women, NRV: nutrient reference value, No.: number, EAR: estimated average requirement, Eq.: equivalents, Vit: vitamin, AI: adequate intake, DHA: docosahexaenoic acid, EPA: eicosapentaenoic acid, DPA: docosapentaenoic acid, g: gram, mg: milligram, µg: microgram. ^Ɨ^ With the exception of magnesium, which is the only nutrient that differs in recommended intake between the age ranges of 19–30 years and 31–50 years in pregnancy. The calculated value of the percentage of women meeting the relevant NRV for magnesium takes into consideration the difference in the EAR as appropriate to each participant’s age bracket [20]. ^¶^ There is no AI, EAR or recommended dietary intake (RDI) for carbohydrates for pregnancy. It is instead recommended that intake be generally increased due to increased physiological demands. This comparative value is based on the ISDS advice of 180 g CHO/day.

**Table 2:** Mean and Range of Serves of Food Groups Consumed by Women and Percentage of Women that Met the AGHE Food Group Serving Recommendations of Pregnant Women Aged 19–50 [17].

Food Group	No. Serves Mean ± SD	Range: Min–Max	% within ± 1 Serve of AGHE Recommendations	% Exceeding AGHE Recommendations	% below AGHE Recommendations
Grain (Cereal)	9.1 ± 4.6	1.2–17.4	28%	50%	22%
Vegetables and Legumes/Beans	5.5 ± 2.4	1.0–11.1	56%	28%	17%
Fruit	2.1 ± 1.4	0.0–5.1	67%	22%	11%
Milk, Yoghurt, Cheese and/or Alternatives	4.5 ± 3.6	1.3–14.1	28%	61%	11%
Meats and/or Alternatives ^§^	5.8 ± 2.9	1.7–13.5	22%	67%	11%

No.: number, SD: standard deviation, Min: minimum, Max: maximum, % percentage of women, AGHE: Australian Guide to Healthy Eating (for Pregnancy). ^§^ This includes meats, poultry, fish, eggs, tofu, nuts and seeds and legumes/beans/tofu.

## Data Availability

Data generated or analysed during this study are available from the corresponding author upon reasonable request.
